# Zaxinone mimics (MiZax) efficiently promote growth and production of potato and strawberry plants under desert climate conditions

**DOI:** 10.1038/s41598-023-42478-3

**Published:** 2023-10-14

**Authors:** Jian You Wang, Muhammad Jamil, Turki S. AlOtaibi, Mohamed E. Abdelaziz, Tsuyoshi Ota, Omer H. Ibrahim, Lamis Berqdar, Tadao Asami, Magdi Ali Ahmed Mousa, Salim Al-Babili

**Affiliations:** 1 The BioActives Lab, Center for Desert Agriculture, King Abdullah University of Science and Technology (KAUST), Jeddah, Saudi Arabia; 2 Department of Agriculture, Faculty of Environmental Sciences, King Abdulaziz University (KAU), 21589 Jeddah, Saudi Arabia; 3 Department of Vegetable Crops, Faculty of Agriculture, Cairo University, Giza, 12613 Egypt; 4 The National Research and Development Center for Sustainable Agriculture (Estidamah), Riyadh, Kingdom of Saudi Arabia; 5 Applied Biological Chemistry, The University of Tokyo, Tokyo, Japan; 6 Department of Ornamental Crops, Faculty of Agriculture, Assiut University, Assiut, 71526 Egypt; 7 Department of Vegetable Crops, Faculty of Agriculture, Assiut University, Assiut, 71526 Egypt; 8 Plant Science Program, Biological and Environmental Science and Engineering Division, King Abdullah University of Science and Technology (KAUST), Jeddah, Saudi Arabia

**Keywords:** Plant physiology, Field trials, Plant sciences

## Abstract

Climate changes and the rapid expanding human population have become critical concerns for global food security. One of the promising solutions is the employment of plant growth regulators (PGRs) for increasing crop yield and overcoming adverse growth conditions, such as desert climate. Recently, the apocarotenoid zaxinone and its two mimics (MiZax3 and MiZax5) have shown a promising growth-promoting activity in cereals and vegetable crops under greenhouse and field conditions. Herein, we further investigated the effect of MiZax3 and MiZax5, at different concentrations (5 and 10 µM in 2021; 2.5 and 5 µM in 2022), on the growth and yield of the two valuable vegetable crops, potato and strawberry, in the Kingdom of Saudi of Arabia. Application of both MiZax significantly increased plant agronomic traits, yield components and total yield, in five independent field trials from 2021 to 2022. Remarkably, the amount of applied MiZax was far less than humic acid, a widely applied commercial compound used here for comparison. Hence, our results indicate that MiZax are very promising PGRs that can be applied to promote the growth and yield of vegetable crops even under desert conditions and at relatively low concentrations.

## Introduction

According to United Nations Food and Agriculture Organization (FAO), our food production system must be nearly tripled to feed the ever expanding global human population by 2050 (FAO: The World Needs 70% More Food by 2050^1^). Indeed, the rapid growth of the human population, environmental pollution, movement of insect pests, and particularly climate change toward high temperatures and drought are challenges for global food security^2^. In this respect, boosting the total yield of crops under suboptimal conditions is one of the undeniable solutions to solve this urgent issue; However, growth and development of plants mostly depend on nutrients availability in the soil, and are highly hindered by adverse environmental factors, including drought, salinity, or biotic stresses^3–5^. These stresses negatively influence the crops’ health and development, potentially leading to reduced crop yields in the end^6^. Moreover, the limitation in freshwater resources severely influences crops irrigation, while global climate changes inevitably decrease the farming land area and events, such as heat waves, reduce crop productivity^7,8^. High temperatures prevail in many regions around the world, including Saudi Arabia. The employment of biostimulants or plant growth regulators (PGRs), which favorably shorten the growth cycle and maximize crop production, may enhance crop tolerance and enable plants to cope with adverse growth conditions^9^. In this respect, biostimulants and PGRs can be utilized at their optimal concentrations to enhance plant growth and performance^10,11^.

**Figure 1:**
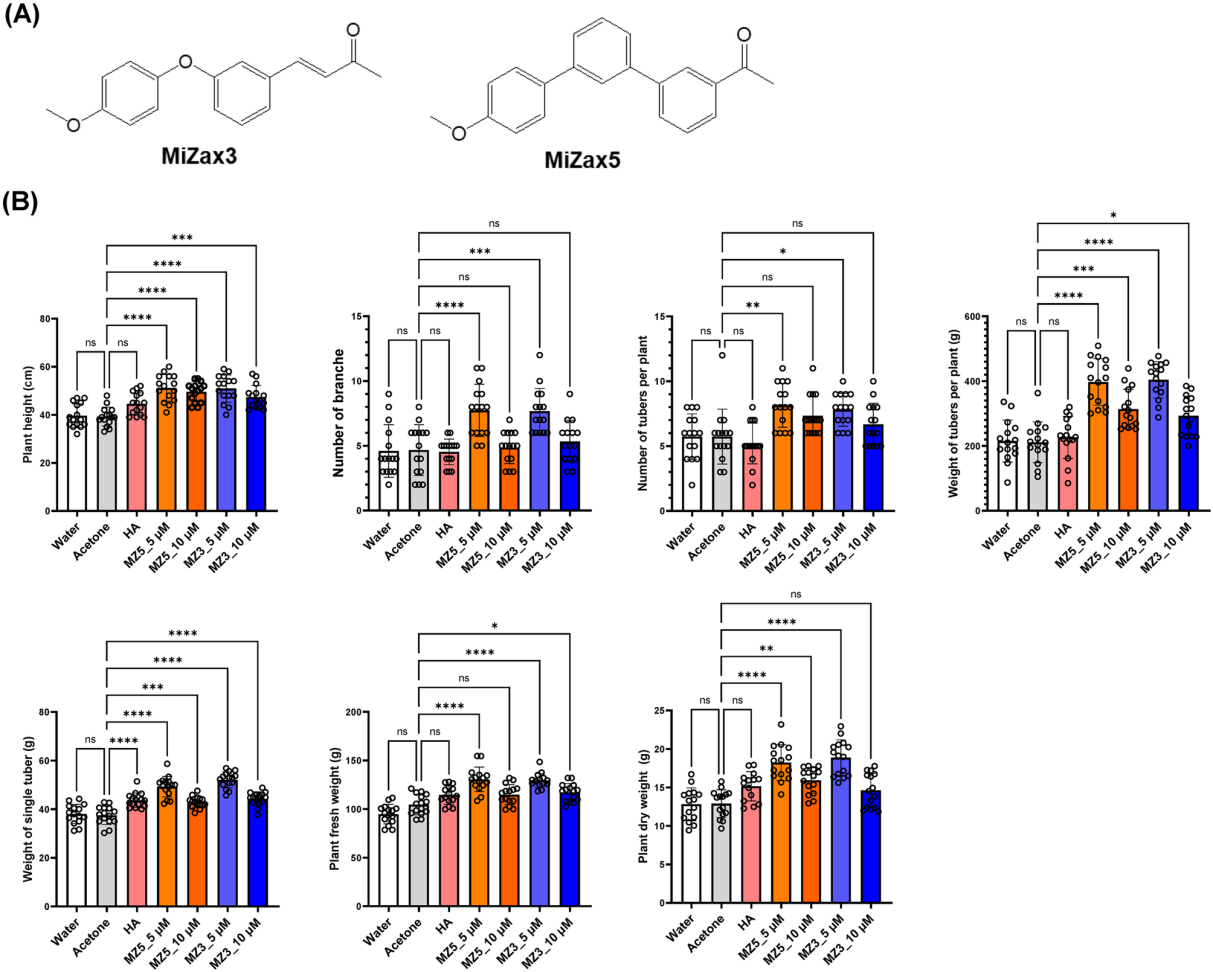
(**A**) Chemical structure of MiZax3 and MiZax5. (**B**) Effect of foliar application of MZ3 and MZ5 at 5 and 10 µM concentrations on potato plants under open field conditions. This experiment was performed in 2021. Data present mean ± SD. n ≥ 15. Statistical analysis was performed using One-way analysis of variance (ANOVA) and Tukey’s post hoc test. Asterisks indicate statistically significant differences as compared to mock (*p < 0.05, **p < 0.01, ***p < 0.001, ****p < 0.0001; ns, non-significant). HA, humic acid; MZ3, MiZax3; MZ5, MiZax5. HA, humic acid; MZ3, MiZax3; MZ5, MiZax5.

Carotenoids are tetraterpenoids that can also act as precursor for the plant hormones abscisic acid (ABA) and strigolactones (SLs)^12–14^ as well as the recently identified growth regulators zaxinone, anchorene, and cyclocitral^15–19^. However, most authentic metabolites, including carotenoid derivatives, are naturally source-restricted and/or instable, which hampers their direct application in the field. Therefore, several ABA and SL analogs/mimics have been developed and tested for agricultural applications in the past few years^20–25^. Similarly, we have recently developed mimics of zaxinone (MiZax), a growth promoting metabolite that exerts its function presumably through enhancing sugar metabolism and regulating SL homeostasis in rice roots^19,26^. The mimics of zaxinone 3 (MiZax3) and MiZax5 (chemical structure shown in Fig. 1A) show biological activities comparable to those of zaxinone with respect to hydroponically and soil grown wild-type rice plants^26^. In addition, treatment of tomato, date palm, green pepper, and squash plants with zaxinone, MiZax3, and MiZx5 enhanced plant growth and performance, i.e. yield quantity and quality in the case of pepper, under greenhouse and open-field conditions, suggesting their utility as biostimulants and PGR^27^. Interestingly, MiZax3 and MiZax5 also increased salt tolerance of green pepper grown under increased salinity and MiZax3 enhanced fruit zinc content when encapsulated with a zinc-containing metal organic frame^7,28^.

In this work, we evaluated three foliar concentrations (5 and 10 µM in 2021 and 2.5 and 5 µM in 2022) of MiZax (MiZax3 and MiZax5) in comparison with the commercial growth-regulator humic acid (HA) on potato (*Solanum tuberosum L*) and strawberry (*Fragaria ananassa*) during 2021 and 2022 in a greenhouse trial with strawberries as well as in four field trials in the Kingdom of Saudi of Arabia, as a classical desert climate place. Although HA is a commonly used biostimulant known for several beneficial effects that include increasing soil nutrients availability and enhancing crop growth by modulating hormone homeostasis^29^, our findings indicate that MiZax was superior to HA in improving crop performance.

## Materials and methods

### Plant material and chemicals

Tubers of the Potato 'Diamond' variety were obtained from Jabbar Nasser Al Bishi Trading Company, Jeddah, Saudi Arabia. Seedlings of the two strawberry varieties 'Sweet Charlie' and 'Festival', and Humic acid were purchased from Modern Agritech company, Riyadh, Saudi Arabia. All plant materials used in this work have complied with the IUCN Policy Statement on Research Involving Species at Risk of Extinction and the Convention on the Trade in Endangered Species of Wild Fauna and Flora.

### Field trials at the KAU research station

#### Field site

The experimental site is located at Hada Al-Sham (21˚48′3′′ N, 39˚43′25′′ E) Al-Jamoom, Saudi Arabia. The soil is sandy loam with pH 7.8 and EC 1.79 dsm^−1^^30^. The soil characteristics are indicated in Supplementary Table S1.

## MiZax effect on strawberry seedlings

Seedlings of strawberry (*Fragaria x ananassa* D. var. Festival) at 3 true leaves stages were arranged in three groups to evaluate the effect of foliar application of 10 µM MiZax3 and MiZax5 on growth characteristics and flowering time under greenhouse conditions. Foliar application with water (containing 0.1% acetone) was used as a mock treatment. MiZax was foliar-sprayed 7 times, at one week interval. Two independent experiments were conducted on 15th and 28th of September 2021, respectively. Application started with 50 mL of each compound then gradually increased up to 250 mL for the last dose. For two weeks continuously, number of flowering plants was recorded daily to calculate the flowering ratio at the beginning of the fourth week. For growth traits, number of leaves per plant, plant fresh and dry weight, total leaf area and number of runners were measured at the end of the growth stage and at the beginning of the generative stage. Leaf area was determined using leaf area meters, while fresh samples were dried in a dry oven for 48h at 100 °C.

### Potato field trails

Two field trails, early and late cultivation, were conducted. Tubers of the potato “Diamond” variety were planted in November and February, for early and late cultivation, respectively. The biostimulants (MiZax-3 and -5) were applied at 5.0 and 10.0 µM concentrations (in 2021) and 2.5 and 5.0 µM (in 2022). Humic acid (HA) at 1g/L were sprayed eight times on weekly basis. Water or acetone were used as negative control. The layout of the field trial is shown in (Supplementary Figure S1). A Randomized Complete Block Design (RCBD) was adopted to establish field trial with plot size measuring 2.5 m × 3.0 m. Each treatment was repeated 3 times as independent replication. Each plot was spaced with 1.0 m and each block spaced with 2.0 m apart. The planting distances were 0.6 m between plants and 1 m between rows. The potato plants were drip irrigated with a 3.4 L per dripper per day. The system was run twice per day for 10 min each, to supply the plants with water. All recommended agricultural practices for growing potatoes under arid conditions were applied^31^. Four months post planting, plant height (cm), number of branches per plant, potato yield components and yield, as well as tubers quality were measured using standard procedure.

### Strawberry field trails

Seedlings of two strawberry varieties (Sweet Charlie and Festival) were tested under field conditions. The biostimulants (MiZax-3 and -5) were applied as foliar spray, at 5.0 and 10.0 µM (in 2021) and 2.5 and 5.0 µM concentrations (in 2022), for eight times on weekly basis. HA at 1g per L was used as foliar spray, parallel to MiZax-3 and -5, blank as H_2_O or acetone was included as a negative control. Strawberry seedlings were planted in early November in 2.5 m × 3 m plots with planting distance of 0.6 m between plants and 1 m between rows. The experiments were carried out in RCBD with 3 replicates. The plants were daily watered at 7 am and 5 pm for 10 min using drip irrigation system containing drippers spaced at 0.6 m and 3.4 L capacity. Agronomic components and yield parameters were measured during the growing season. Fruit quality including TSS (%), Vitamin C^32^, Acidity and total phenols^33^ were performed in the lab of postharvest physiology and technology, King Abdulaziz University.

### Statistical analysis

Data are presented as means and their variations as standard deviation. The statistical significance was determined by one-way analysis of variance (one-way ANOVA) or two-way ANOVA with Tukey’s multiple comparison test, using a probability level of *p* < 0.05, or two-tail student *t*-test with denote significant differences (*p < 0.05, **p < 0.01, ***p < 0.001, ****p < 0.0001). All statistical elaborations were performed using GraphPad Prism, version 8.3.0. Principal component analysis (PCA), a multivariate statistical technique, was conducted to examine links by using the R package^34^.

## Results and discussion

### MiZax enhanced potato plant growth and yield in early and late cropping seasons

In the previous report, we showed growth-improving activities of MiZax on horticultural plants at both 5 and10 µM with an enhancement of Soil Plant Analysis Development (SPAD) chlorophyll metrics^27^. Based on these results, we used the same concentration to assess the effect of MiZax on potato, an important food crop worldwide, in a field trial under desert climate conditions in 2021. In particular, we were interested in testing if MiZax can increase the accumulation of starch, the end product of photosynthesis. In general, application of MiZax improved the growth of potato plants, leading to enhanced plant height, biomass and branching number, in comparison to Humic acid (HA) (Fig. 1B). Moreover, we observed with 5 µM of MiZax3 and MiZax5 a stronger effect in increasing plant height, branch number, and plant biomass, compared to10 µM (Fig. 1B). Consistent with improved growth, MiZax application also increased the yield calculated based on the number and weight of produced tubers. The general positive effect was less pronounced when MiZax were applied at 10 µM concentrations, suggesting that these compounds should be applied below this concentration (Fig. 1B). Moreover, we did not observe differences in all recorded parameters between acetone (mock) and water (control) treatment, which indicates that the observed growth-regulating effects were not caused by the solvent, in line with our previous report^27^.

**Figure 2:**
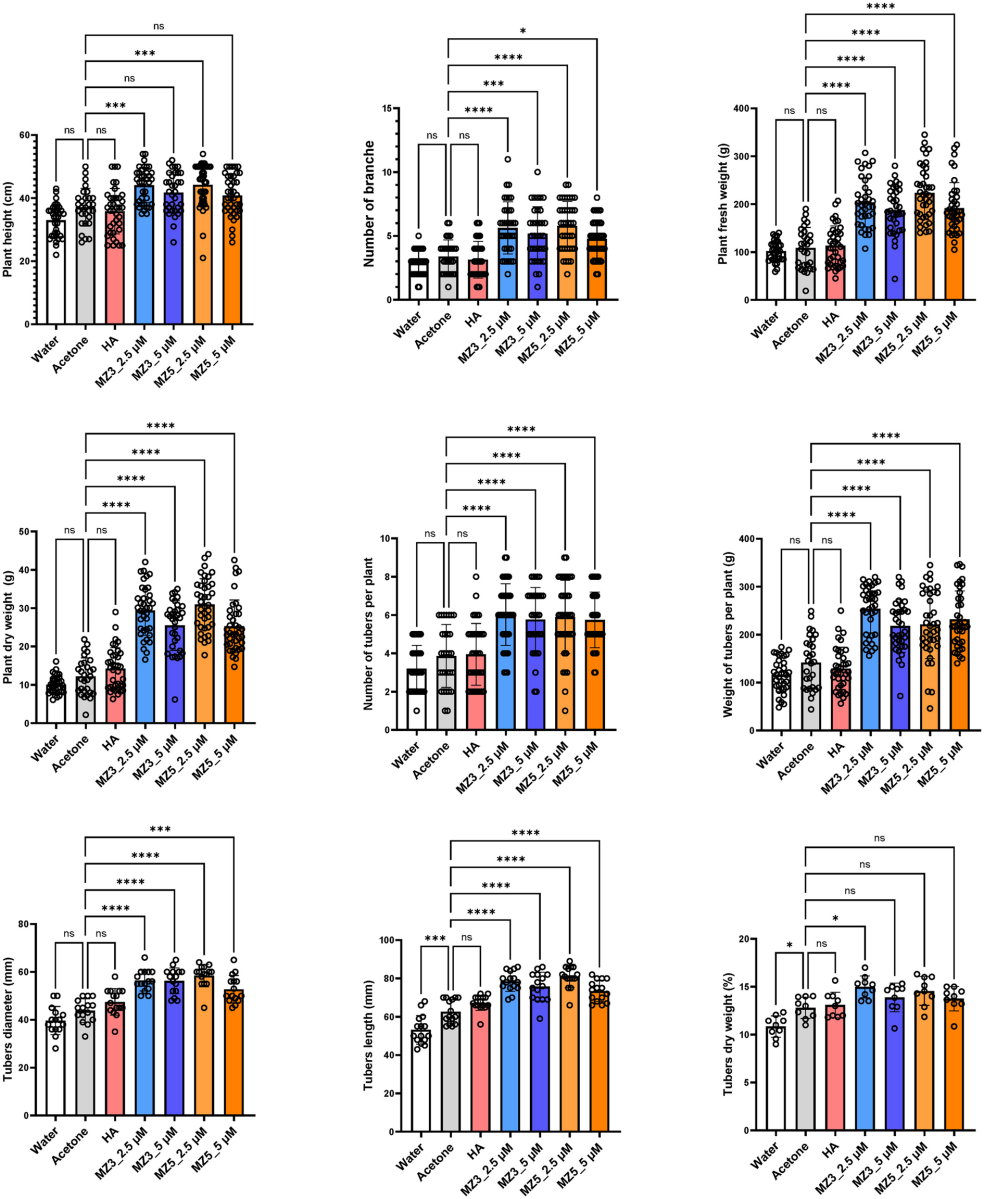
Plant phenotypical evaluation of MiZax effect on early cropping potato plants from the field of KAU, performed in 2022. Data represent mean ± SD. n ≥ 15. Statistical analysis was performed using One-way analysis of variance (ANOVA) and Tukey’s post hoc test. Asterisks indicate statistically significant differences as compared to mock (*p < 0.05, **p < 0.01, ***p < 0.001, ****p < 0.0001; ns, non-significant). HA, humic acid; MZ3, MiZax3; MZ5, MiZax5. HA, humic acid; MZ3, MiZax3; MZ5, MiZax5.

As there are early and late cropping seasons for potato in Saudi Arabia, we performed the second field study in 2022, with the low concentrations (2.5 and 5 µM), in two seasons to assess seasonal effects in open field (Supplementary Figure S2A). As expected, the application of the two MiZax at 5 µM led to a similar growth promoting effect as in the first trial: an increase of plant height, more branches, higher biomass, and number of tubers (Fig. 2; Supplementary Figure S3). Importantly, we observed substantial effect of these PGRs already at 2.5 µM, while HA treatment did not show the assumed impact. This result suggests that MiZax may be even applied at a concentration lower than expected. Furthermore, the application of MiZax also increased the length and width of the tubers (Supplementary Figure S2B). We also detected a significant increase in the weight of tubers; however, only upon the application of 2.5 µM concentration in both cropping seasons.

**Figure 3:**
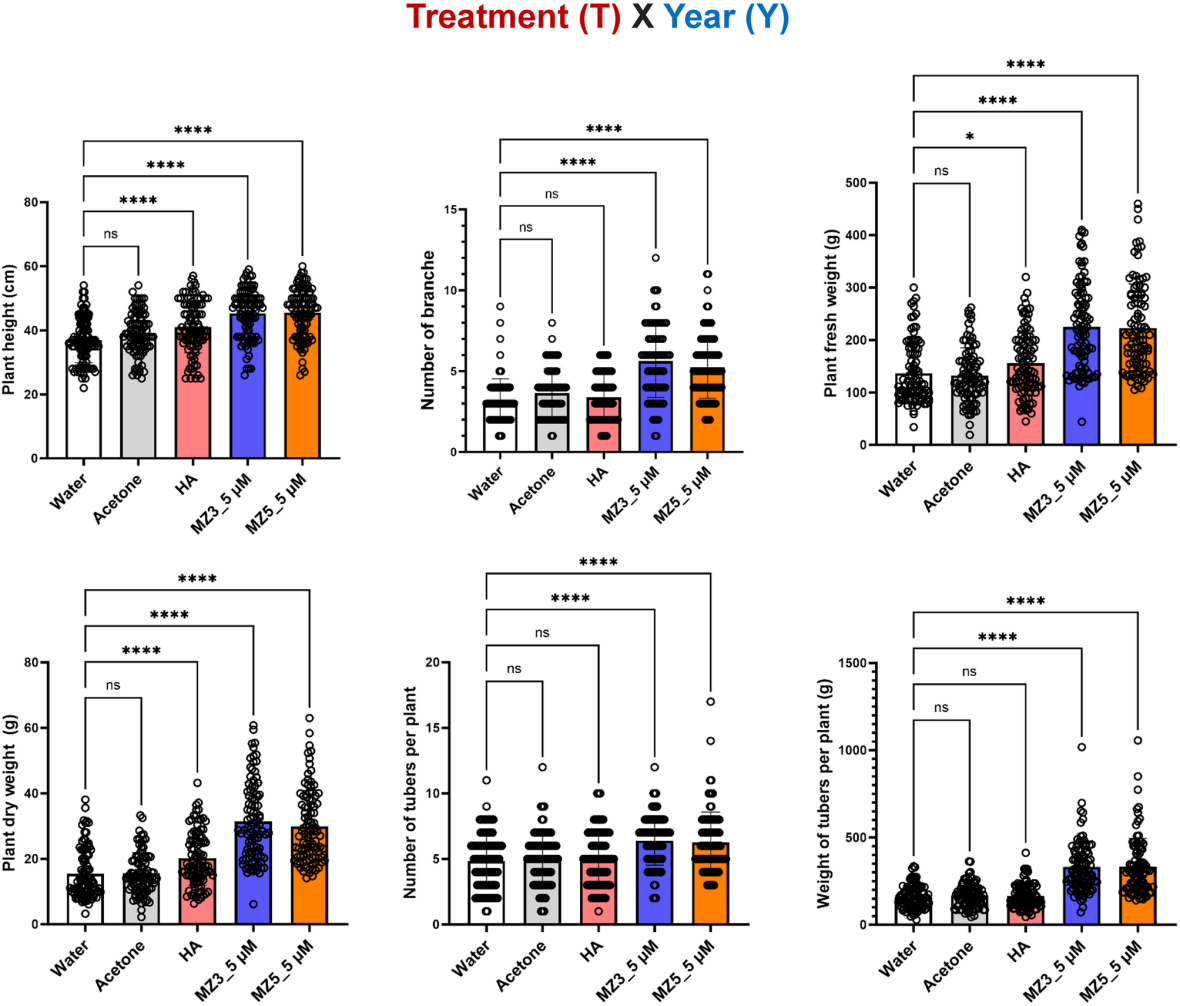
Examine the influence of the treatment (T) at 5 µM, the year (Y), and their interaction (T x Y) for potatoes. Data represent mean ± SD. n ≥ 30. Statistical analysis was performed using Two-way analysis of variance (ANOVA). Asterisks indicate statistically significant differences as compared to mock (*p < 0.05, **p < 0.01, ***p < 0.001, ****p < 0.0001; ns, non-significant). HA, humic acid; MZ3, MiZax3; MZ5, MiZax5.

To further understand the influence of the treatment (T) and the year (Y), two-way ANOVA was employed to examine their interaction (T x Y). Although all the biostimulants (T) remarkably increased the height and biomass of potato plants, only MiZax3 and MiZax5 significantly promoted the number and weight of tubers, indicating that the two-way response on potato tubers to both MiZax was substantially similar (Fig. 3). Additionally, in early season, the weather (https://www.timeanddate.com/weather/saudi-arabia/jeddah/climate) was hotter (median 32 °C and 52% humidity at 2022) than the second one (averaged 28 °C and 52% humidity at 2022 ), which remarkably reduced the general biomass of tubers (Fig. 2; Supplementary Figure S3).

Nevertheless, MiZax treatment still showed a tendency towards promoting growth of potato plants grown on late cropping season. Overall, our three independent experiments inarguably demonstrated that the application of MiZax has a significant effect on plant architecture, by increasing the number of branches. Indeed, there were significant two-way interaction effects between (T) with (Y) on the branch numbers upon MiZax treatments (Fig. 3). This result is in agreement with their activity as negative regulator of strigolactone (SL) biosynthesis^26^. In addition, we previously showed that zaxinone treatment triggered starch accumulation in rice roots^35^, which may explain the enhanced potato tuber size and weight after MiZax treatment as the composition of tubers is mainly starch.

### Field application of MiZax improved growth and yield of strawberry in desert conditions

Fruit crops are economically important plants. Strawberry is sensitive to abiotic stress conditions, such as drought and heat. Hence, we explored the effect of MiZax on strawberry, using foliar application. We first supplied MiZax at a 10 µM concertation to assess its impact on the growth of strawberry (cv Festival). Interestingly, we observed that MiZax3 significantly increased the number of runners, which corresponds to increased branching, while MiZax5 improved the flowering ratio, plant biomass, and leaf areas under greenhouse conditions (Supplementary Figure S4), indicating the two compounds may differ in their biological activities^26,27^. To get deeper insights into their effects on strawberry in a real farming environment, we conducted a pilot field trail by applying 5 and 10 µM of MiZax to strawberry plants (cv Sweet Charlie) grown in semi-sand soil (Figure S5A) in 2021. We did not observe an increase in plant biomass, in comparison to HA, but detected a tendency to enhanced fruit number (Figure S6A-B). Nevertheless, MiZax application caused a significant increase in the weight per fruit with a hint on concentration dependency (Supplementary Figure S5B; Supplementary Figure S6B), suggesting that these PGRs have a positive impact on the strawberry fruit quality, when applied under desert conditions.

**Figure 4:**
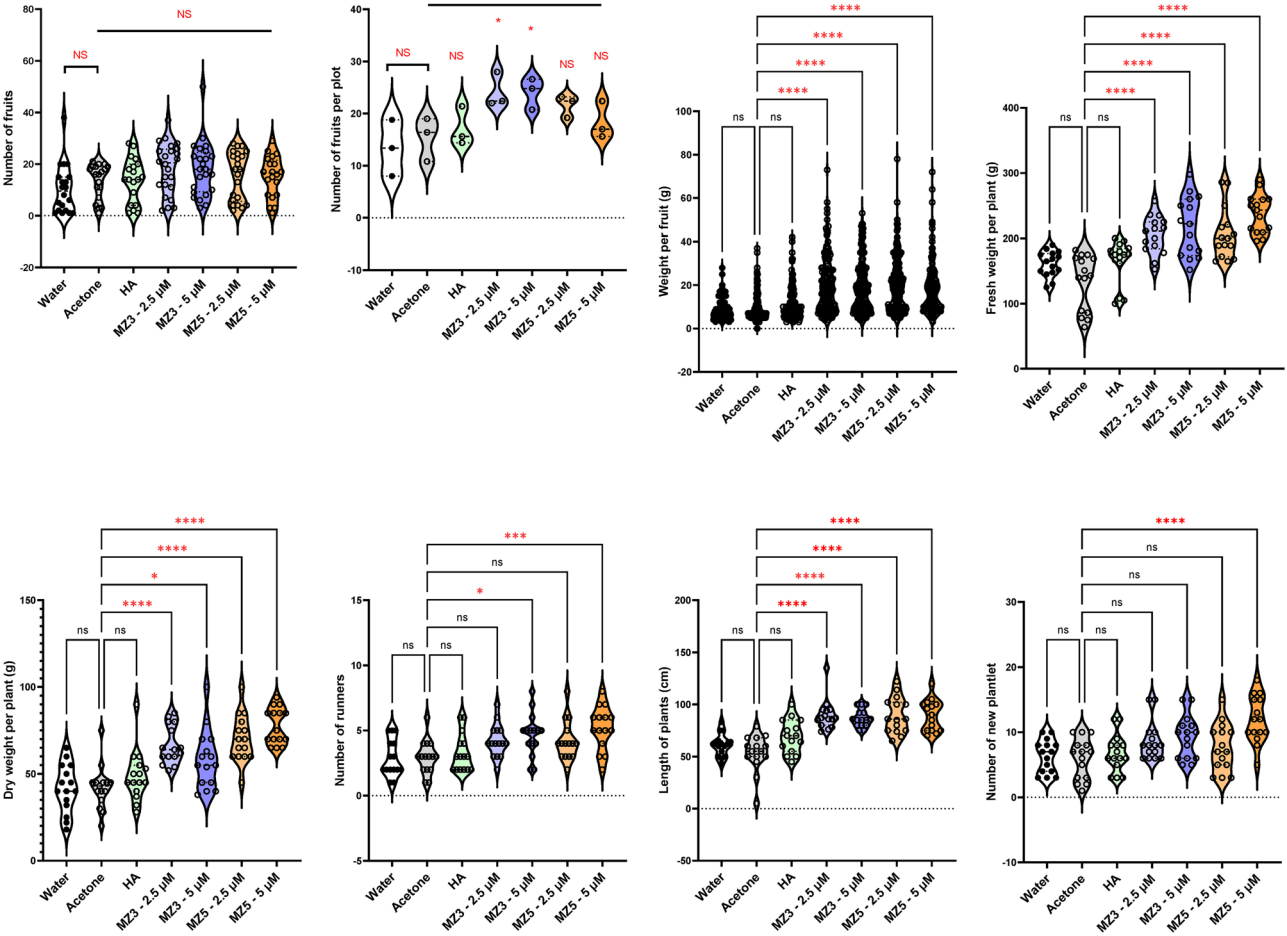
MiZax effect on plant architecture and fruit production of strawberry (cv Sweet Charlie) from the field of KAU, performed in 2022. Data represent mean ± SD. n ≥ 15, except fruit per plot was calculated from the average from 15 plants of three plots (n = 3). Statistical analysis was performed using One-way analysis of variance (ANOVA) and Tukey’s post hoc test or using two-tail student *t*-test. Asterisks indicate statistically significant differences as compared to mock (*p < 0.05, **p < 0.01, ***p < 0.001, ****p < 0.0001; ns, non-significant). HA, humic acid; MZ3, MiZax3; MZ5, MiZax5.

To understand if the growth-improving effect depends on the type of cultivars, we selected the two commercially used strawberry varieties (Sweet Charlie and Festival) in Saudi Arabia for two field studies conducted in 2022, using the low MiZax concentrations (2.5 and 5 µM). In Sweet Charlie variety, although the total number of fruits did not increase significantly, the fruit biomass was generally higher in MiZax-treated plants, and the fruit number per plot increased upon MiZax3 treatment (Fig. 4). These data indicate again that MiZax3 and MiZax5 may differ in their biological activity. Moreover, we observed an increase in plant fresh and dry weight as well as in the shoot length of plants upon MiZax treatment. With respect to the number of runners and new plantlets, we detected an enhancement only with 5 µM application of MiZax (Fig. 4), which suggested that the optimal concertation of MiZax depends on plant species.

**Figure 5:**
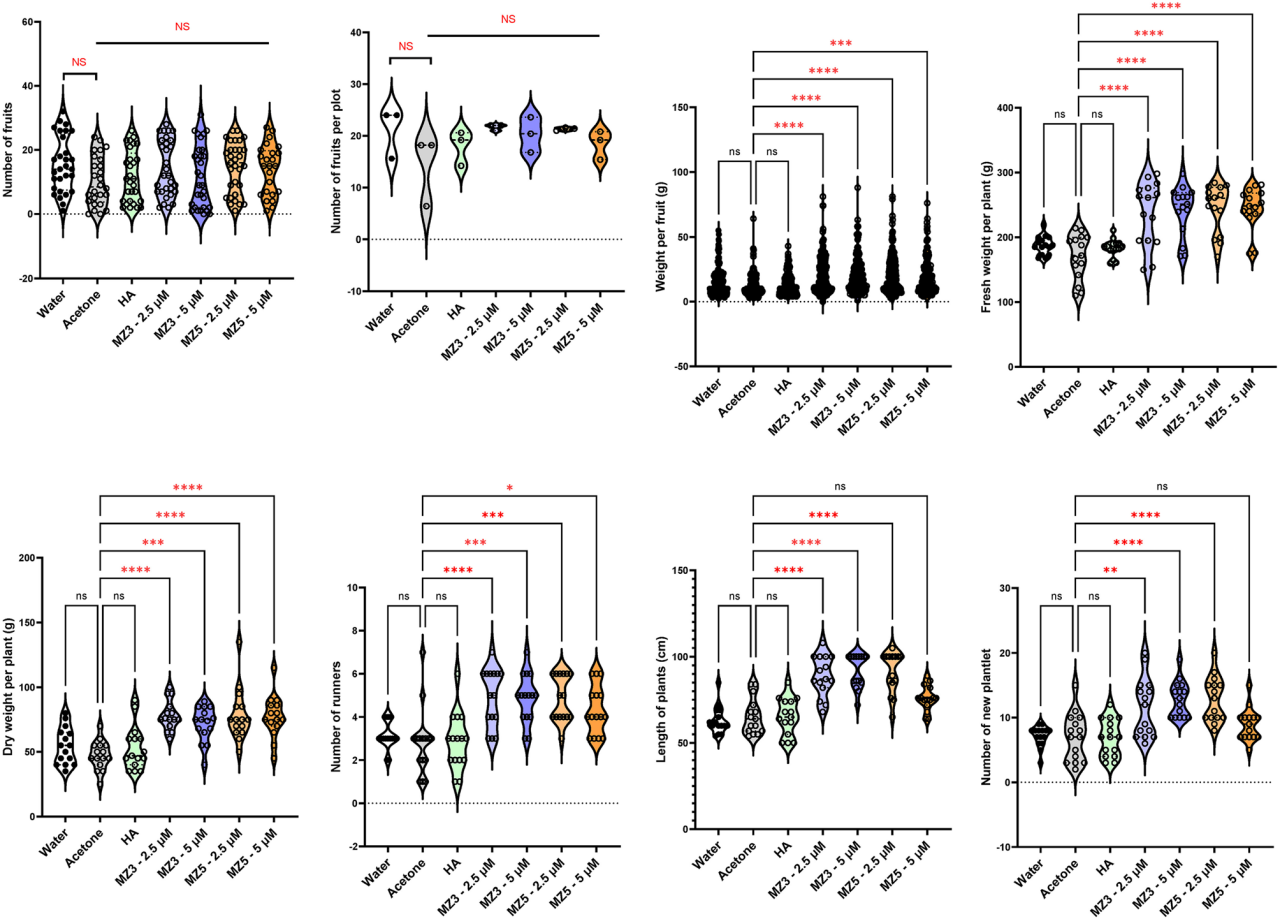
MiZax effect on plant architecture and fruit production of strawberry (cv Festival) from the field of KAU, performed in 2022. Data represent mean ± SD. n ≥ 15, except fruit per plot was calculated from the average from 15 plants of three plots (n = 3). Statistical analysis was performed using One-way analysis of variance (ANOVA) and Tukey’s post hoc test or using two-tail student *t*-test. Asterisks indicate statistically significant differences as compared to mock (*p < 0.05, **p < 0.01, ***p < 0.001, ****p < 0.0001; ns, non-significant). HA, humic acid; MZ3, MiZax3; MZ5, MiZax5.

We also observed similar growth-promoting activities in the Festival variety of strawberry, with respect to fruit weight and plant biomass (Fig. 5); however, we did not detect significant difference in the total number of fruits per plant or of fruits per plot (Fig. 5). Interestingly, MiZax application enhanced plant’s length and the number of runners, suggesting the utility of these PGRs for improving the growth of fruit crops (Fig. 5). In addition, we measured several biochemical parameters to access the fruit quality of the two varieties harvested from the field, but we did not get any difference among all treatments (Supplementary Figure S7; Supplementary Figure S8).

**Figure 6:**
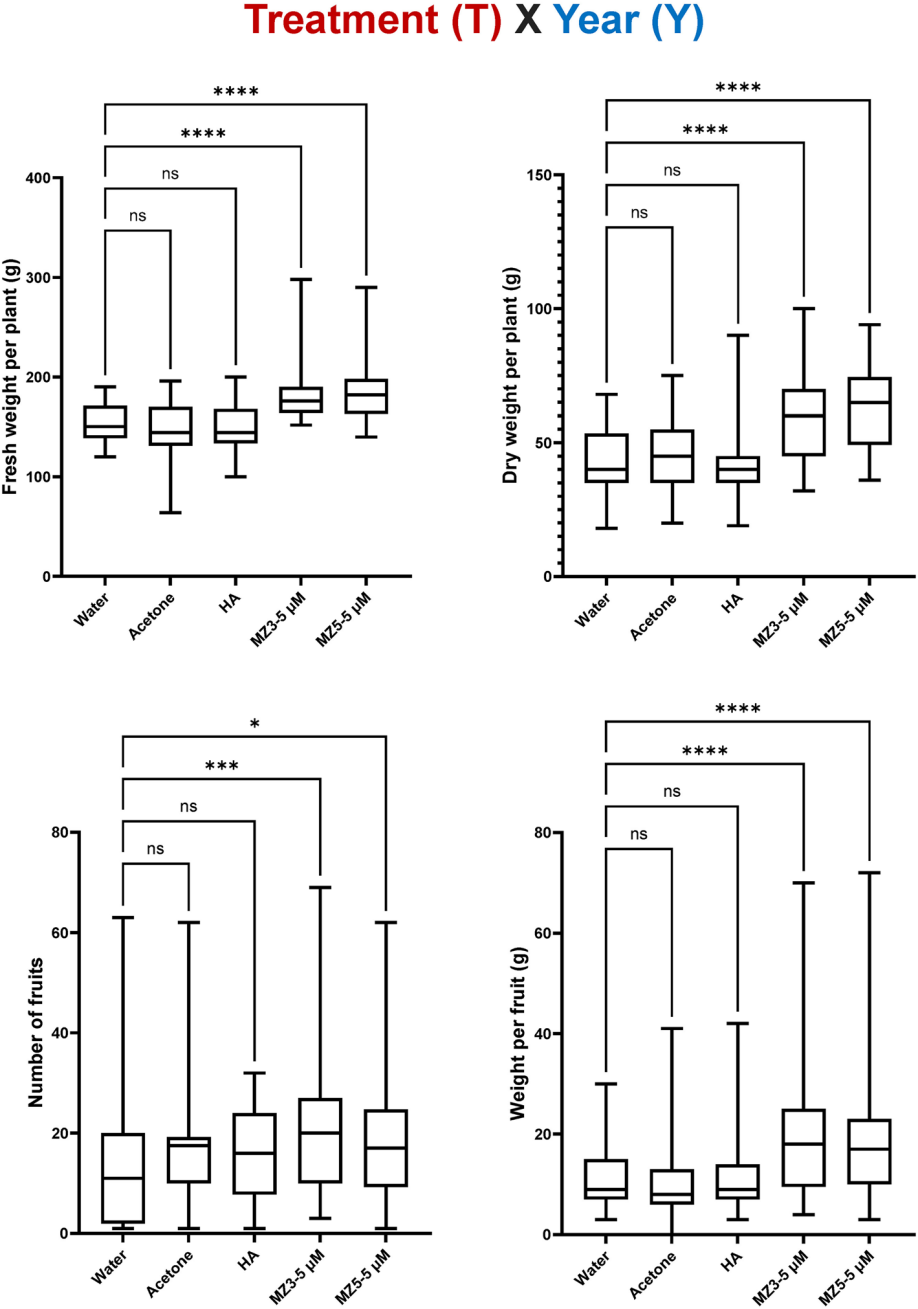
Evaluation on the influence of the 5 µM treatment (T), the year (Y), and their interaction (T x Y) for strawberry (cv Sweet Charlie). Data represent mean ± SD. n ≥ 30. Statistical analysis was performed using Two-way analysis of variance (ANOVA). Asterisks indicate statistically significant differences as compared to mock (*p < 0.05, **p < 0.01, ***p < 0.001, ****p < 0.0001; ns, non-significant). HA, humic acid; MZ3, MiZax3; MZ5, MiZax5.

It seems that MiZax3 and MiZax5 differed in their biological activities in our strawberry studies. We firstly investigated the influence of the treatment (T) and the year (Y) on the same cultivar (Sweet Charlie), using two-way ANOVA to determine their interaction (T x Y). Consistently, HA did not show any effect on the strawberry cultivar (Sweet Charlie), while 5 µM MiZax3 and MiZax5 significantly improved the biomass of plants and fruits (Fig. 6), suggesting that the two-way interaction of both MiZax was considerably similar in improving plant growth of strawberry.

**Figure 7:**
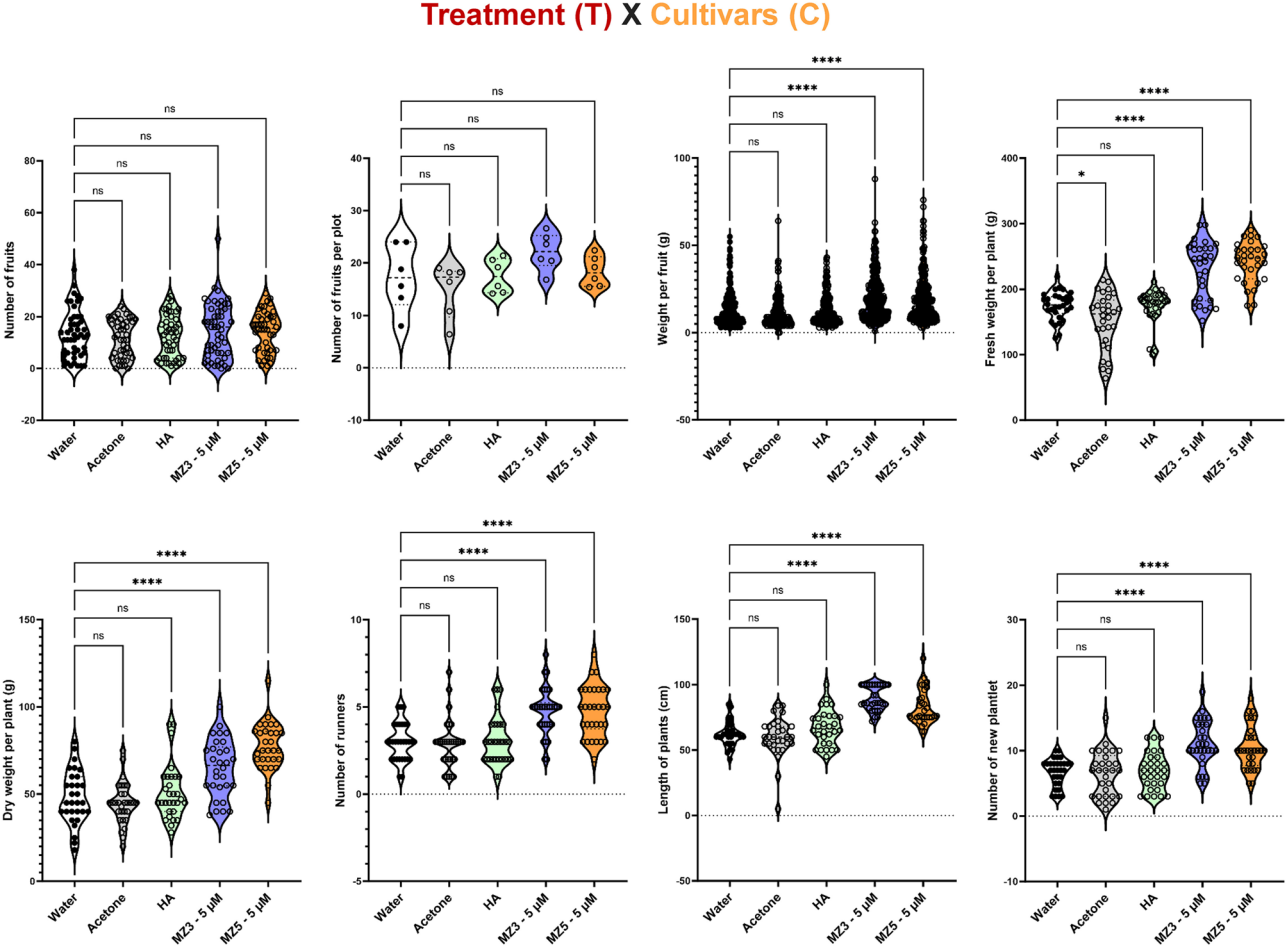
Assessment of the 5 µM treatment (T), the two cultivars (C), and their interaction (T x C) for strawberry. Data represent mean ± SD. n ≥ 30, except fruit per plot was calculated from the average from 15 plants of three plots (n = 6). Statistical analysis was performed using Two-way analysis of variance (ANOVA). Asterisks indicate statistically significant differences as compared to mock (*p < 0.05, **p < 0.01, ***p < 0.001, ****p < 0.0001; ns, non-significant). HA, humic acid; MZ3, MiZax3; MZ5, MiZax5.

Moreover, considering that MiZax showed slight differences in their activity on the two cultivars (Fig. 4; Fig. 5), we performed a two-way ANOVA analysis that compared the treatment (T) and the two cultivars (C). First of all, number of fruits per plot was not affected by any treatment (Fig. 7), suggesting that there was no clear interaction between (T x C) and implying that neither MiZax nor HA promoted the numbers of fruits in general. In contrast, MiZax, but not HA, significantly improved plant weight, fruit weight, runners, and new plantlets (Fig. 7), indicating that MiZax3 and MiZax5 notably promoted the growth of strawberry plants from different cultivars. Based on (T x Y) and (T x C) two-way ANOVA analysis, we can conclude that the growth-promoting activities of MiZax3 and MiZax5 were very similar and consistent under field conditions.

**Figure 8:**
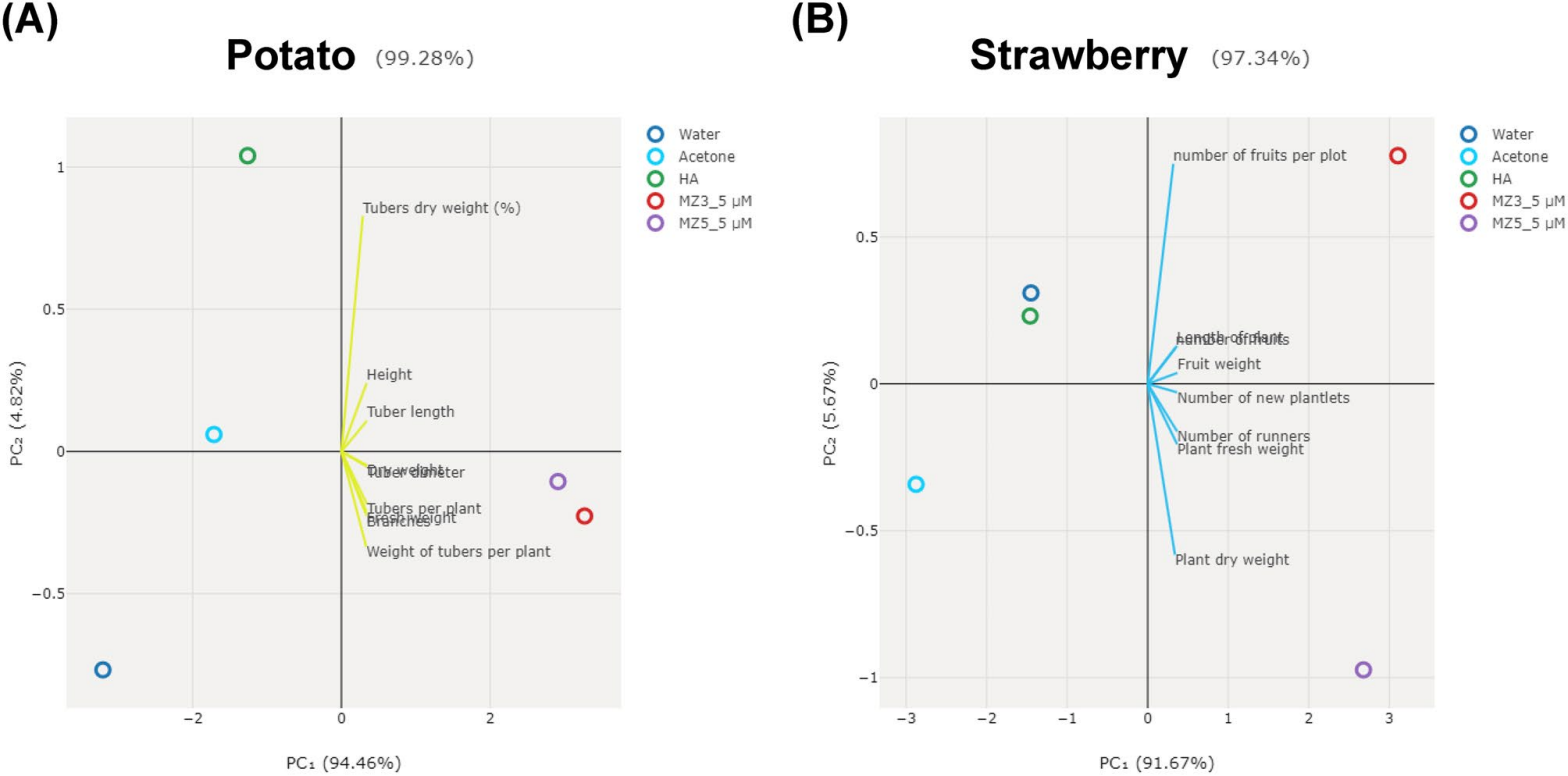
Principal component analysis (PCA) of (A) potato (T x Y) and (B) strawberry (T x C). The scores plot for both groups the lines connecting each components lead to the cluster center.

Finally, we used Principal Components Analysis (PCA) to assess the effects of applied compounds on potato (T x Y) and strawberry (T x C). The plots indicated that HA treatments were similar to either acetone in potato or water in strawberry (Fig. 8), revealing a relative minor positive impact on plant growth. Interestingly, the overall effects of MiZax3 and MiZax5 showed same distributions on the potato (Fig. 8A), while there were different distributions between these two compounds in Strawberry (Fig. 8B). Although both MiZax3 and MiZax5 showed major positive distributions on plant growth and yields, PCA analysis suggested that the growth-regulatory activities might also depend on plant species.

## Conclusion

Taken together, based on our five independent field studies with two valuable crops, and in agreement with our previous reports^26,27^ from 2020 to 2022, MiZax3 and MiZax5 are promising PGRs that improve the performance and production of several crop plants, including a cereal, a tree plant (date palm), and horticultural fruit crops^26,27^. Although the molecular mechanism beyond their biological activities remains elusive, they have large potential for field application. Most importantly, the applied amounts of MiZax were much lower (at the micro-molar or mg level) and the positive effects much more pronounced, compared to humic acid. Thus, we estimate the amount of MiZax3 per application (from low to high concentration): 3, 6, or 12 g and of MiZx5: 4, 7, or 13 g per hectare, which makes the employment of these PGRs for improving crop performance quite feasible.

### Supplementary Information

Supplementary Information.

## Data Availability

All data generated or analyzed in this study are included in this published article and its supplementary information files.
