# Supplementary material for: Zaxinone mimics (MiZax) efficiently promote growth and production of potato and strawberry plants under desert climate conditions
Source: Sci Rep. 2023 Oct 14;13:17438. doi: 10.1038/s41598-023-42478-3 (PMC10576822; doi:10.1038/s41598-023-42478-3)
Supplement: Supplementary file 1 — Supplementary Information. [file 41598_2023_42478_MOESM1_ESM.pdf]

Table S1. Soil physical and chemical proprieties of the KAU Agriculture Research Station at Hada AlSham, Al-Jamoom, Saudi Arabia.

pH (unit)	EC (ds/m)	Sandy Loam Soil particle size (%)			Organic matter (%)	Organic carbon (%)	Available macro nutrients (%)		
		Sand	Silt	Clay			N	P	K
7.83	1.79	84.21	14.05	1.74	0.453	0.500	0.215	0.070	0.781

Total elements (mg/kg)

Cr	Pb	Ni	Cd	Mn	Fe	Ca (%)	Mg (%)	Cu	Zn	Na (%)
0.11	4.21	0.52	0.06	144.44	239.40	1.38	1.15	4.78	32.98	0.14

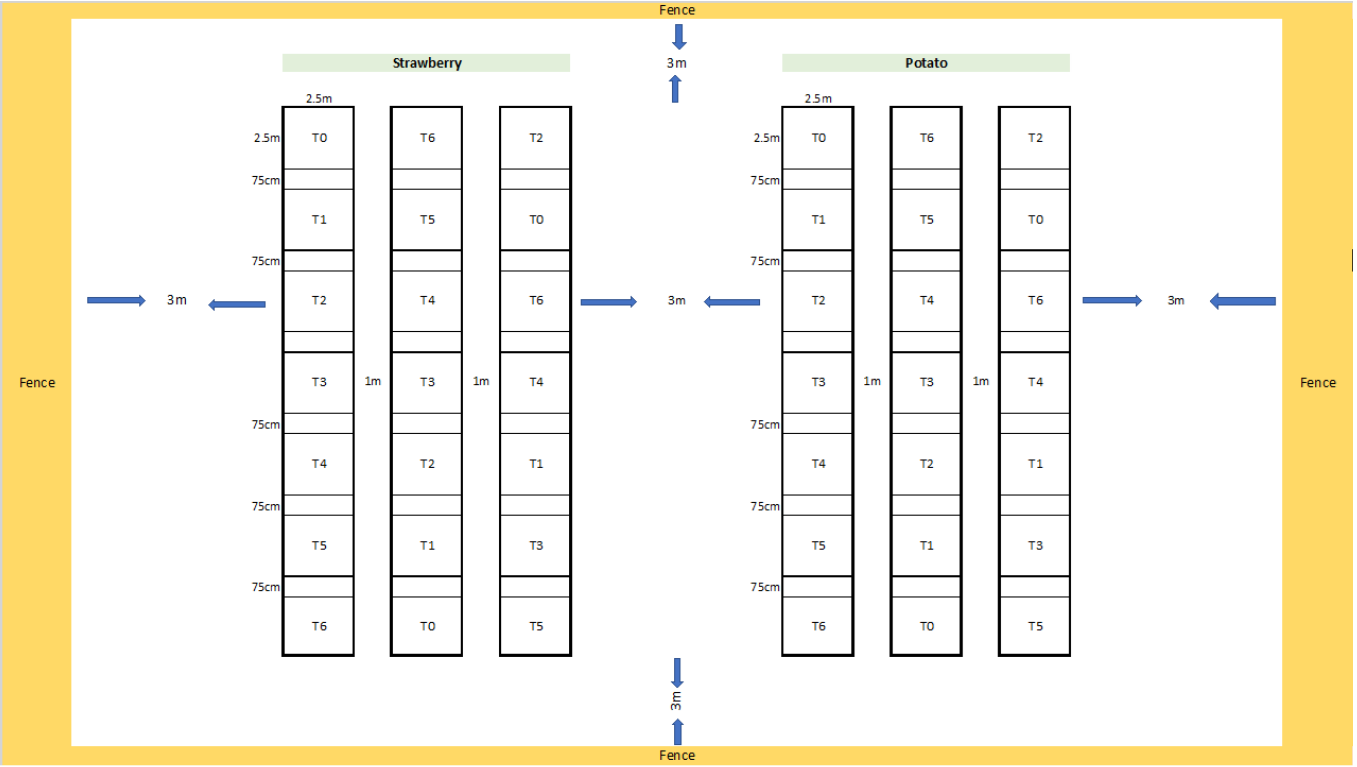

Starwberry

- Space between plots = 1m
- Plot size = 2.5m x 2.5m
- No. of tubes per plot = 4
- Total no. of plants per plot= 16
- Total no. of plots = 21

Potato

- Space between plots = 1m
- Plot size = 2.5m x 2.5m
- No. of tubes per plot = 4
- Total no. of plants per plot= 16
- Total no. of plots = 21

Figure S1. Experimental field layout in KAU station.

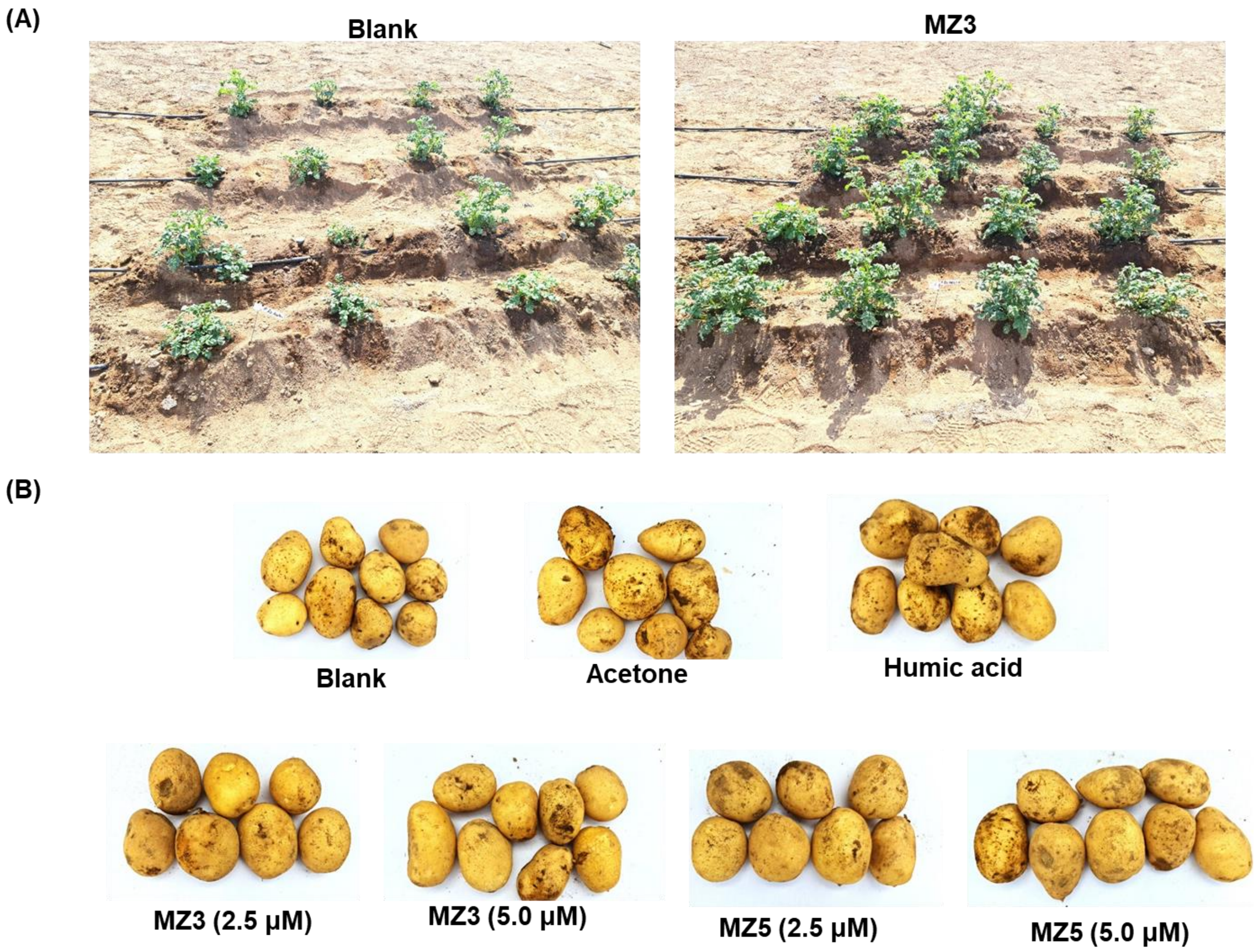

Figure S2. (**A**). Field pictures of potato plants treated with acetone or MiZax3 under desert conditions. (**B**). Pictures of potatoes harvested from different treatment. HA, humic acid; MZ3, MiZax3; MZ5, MiZax5.

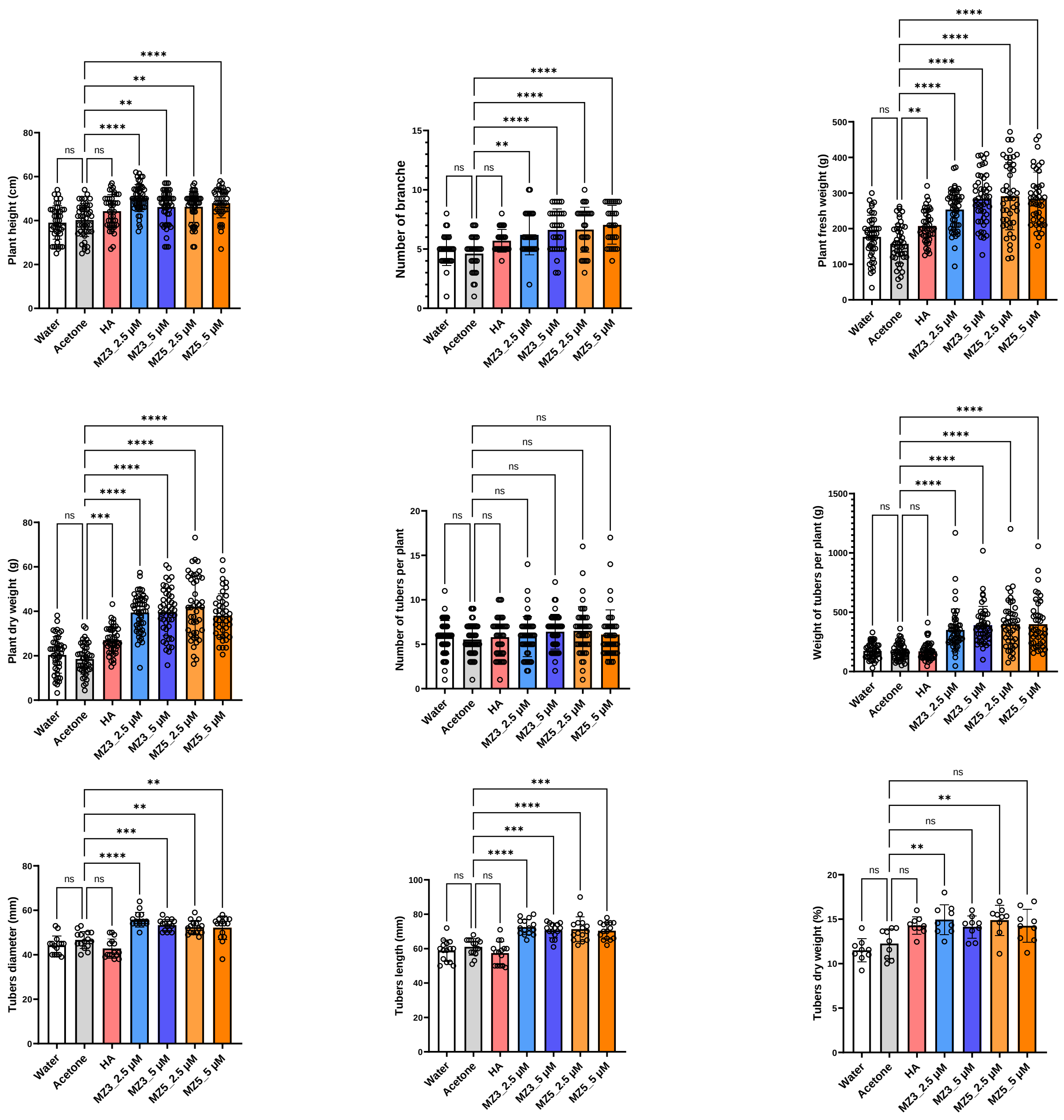

Figure S3. Phenotyping of MiZax effect on late cropping potato plants from the field of KAU, performed in 2022. Data represent mean ± SD. n ≥15. Statistical analysis was performed using One-way analysis of variance (ANOVA) and Tukey's post hoc test. Asterisks indicate statistically significant differences as compared to mock (**p* < 0.05, ***p*< 0.01, ****p*< 0.001, *****p*< 0.0001; ns, non-significant). HA, humic acid; MZ3, MiZax3; MZ5, MiZax5. HA, humic acid; MZ3, MiZax3; MZ5, MiZax5.

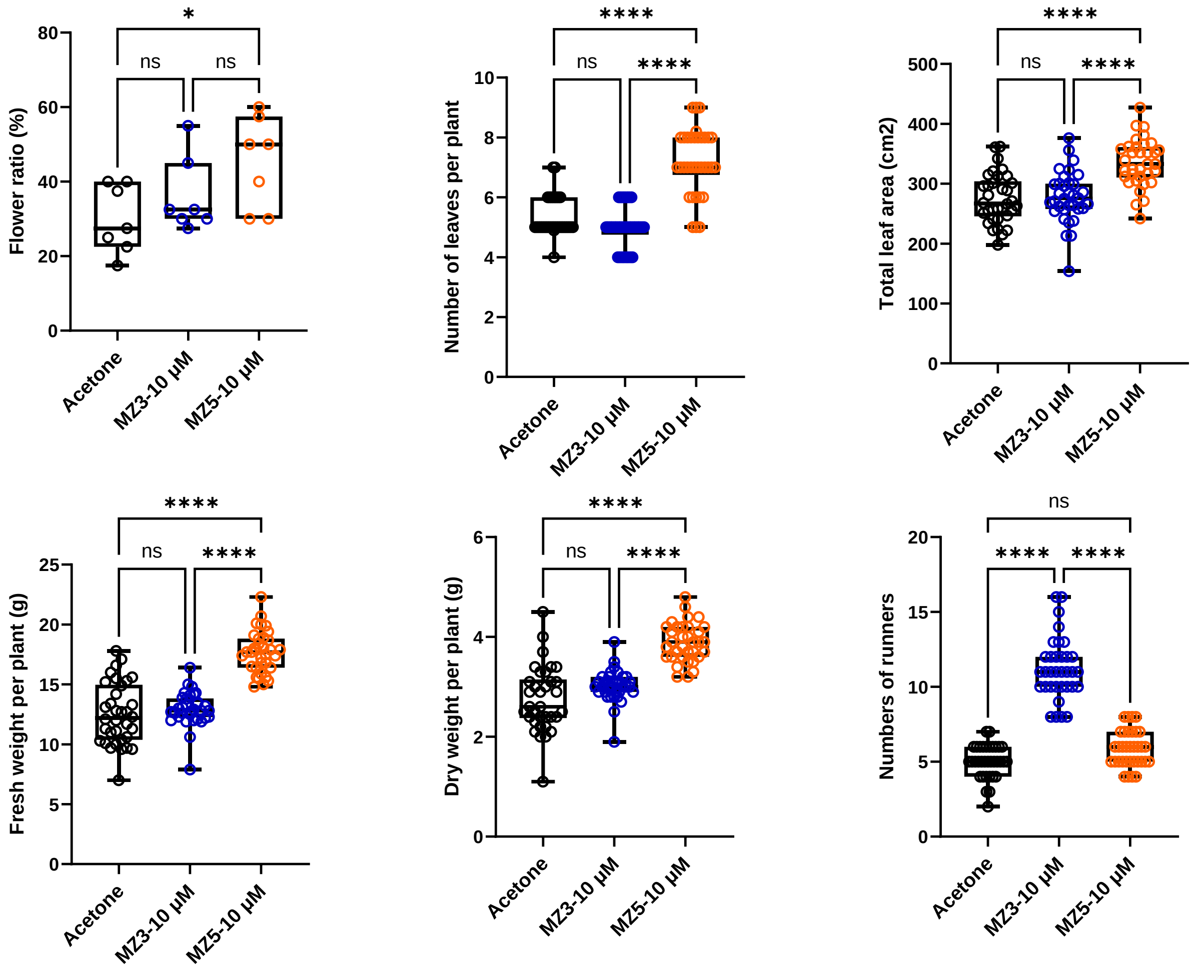

Figure S4. MiZax effect on strawberry (*Fragraria X ananssa Duch* cv Festival) grown under greenhouse conditions, performed in 2021. Data represent mean ± SD. n =34. Statistical analysis was performed using One-way analysis of variance (ANOVA) and Tukey's post hoc test. Asterisks indicate statistically significant differences as compared to mock (*p < 0.05, **p< 0.01, ***p< 0.001, ****p< 0.0001; ns, non-significant). MZ3, MiZax3; MZ5, MiZax5.

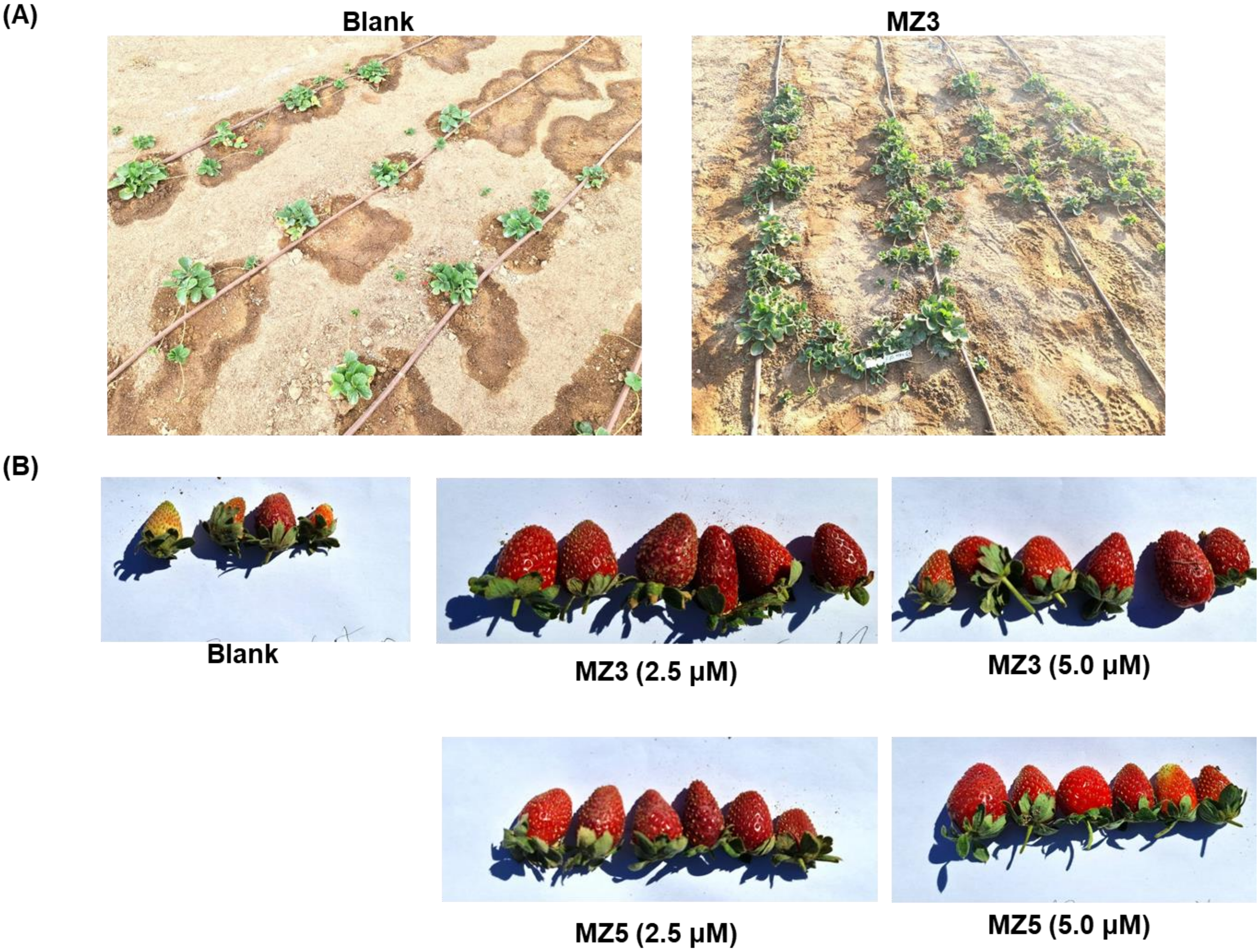

Figure S5. (**A**). Field pictures of strawberry plants grown under desert conditions. (**B**). Pictures of harvested strawberry among different treatment. HA, humic acid; MZ3, MiZax3; MZ5, MiZax5.

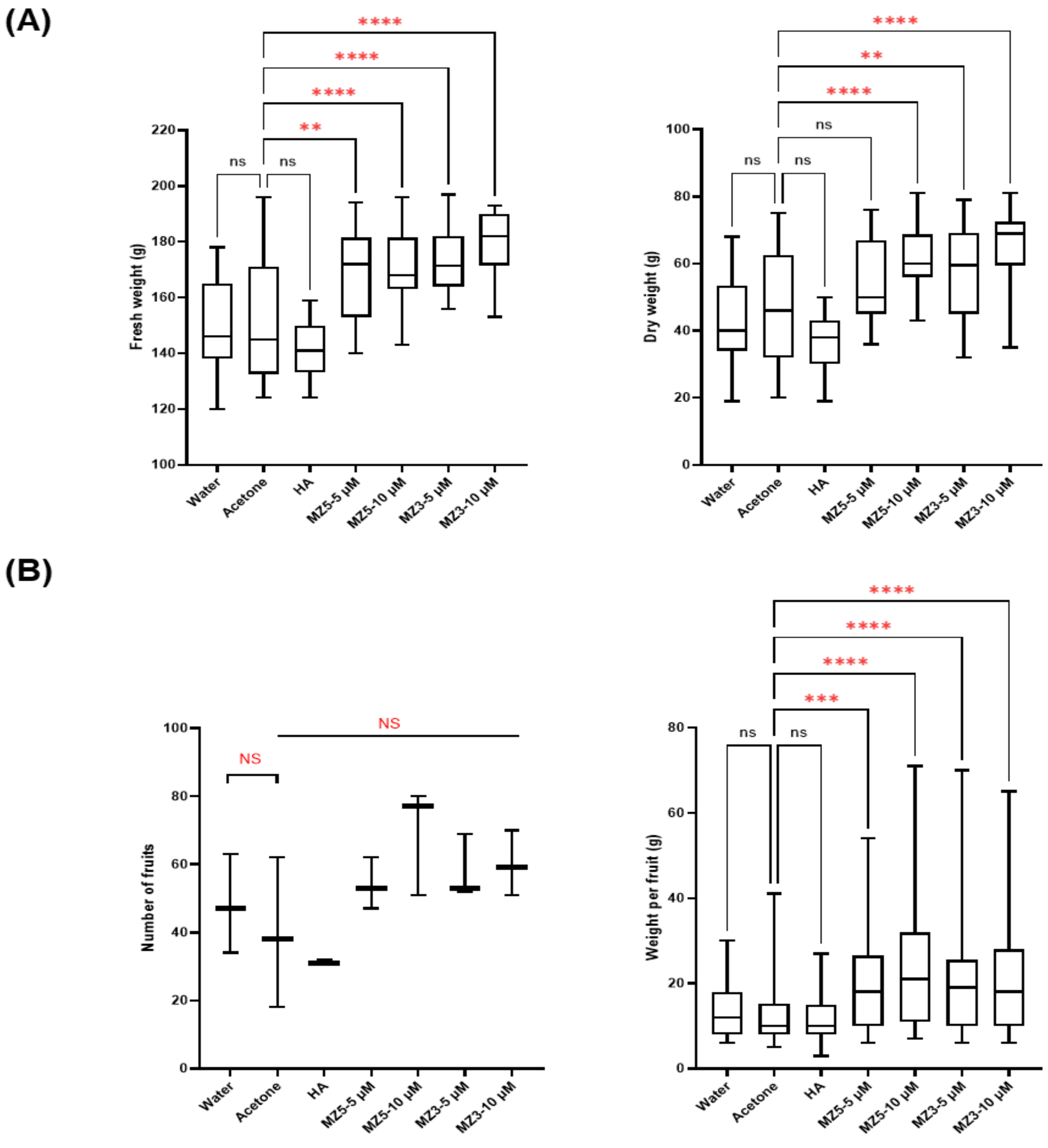

Figure S6. (**A**). Phenotyping of strawberry plants (cv Sweet Charlie) treated with MiZax in KAU field, performed in 2021. (**B**). Evaluation of strawberry fruits upon MiZax treatment. Data represent mean ± SD. n ≥10. Statistical analysis was performed using One-way analysis of variance (ANOVA) and Tukey's post hoc test or using two-tail student *t*-test. Asterisks indicate statistically significant differences as compared to mock (**p* < 0.05, ***p*< 0.01, ****p*< 0.001, *****p*< 0.0001; ns, non-significant). HA, humic acid; MZ3, MiZax3; MZ5, MiZax5.

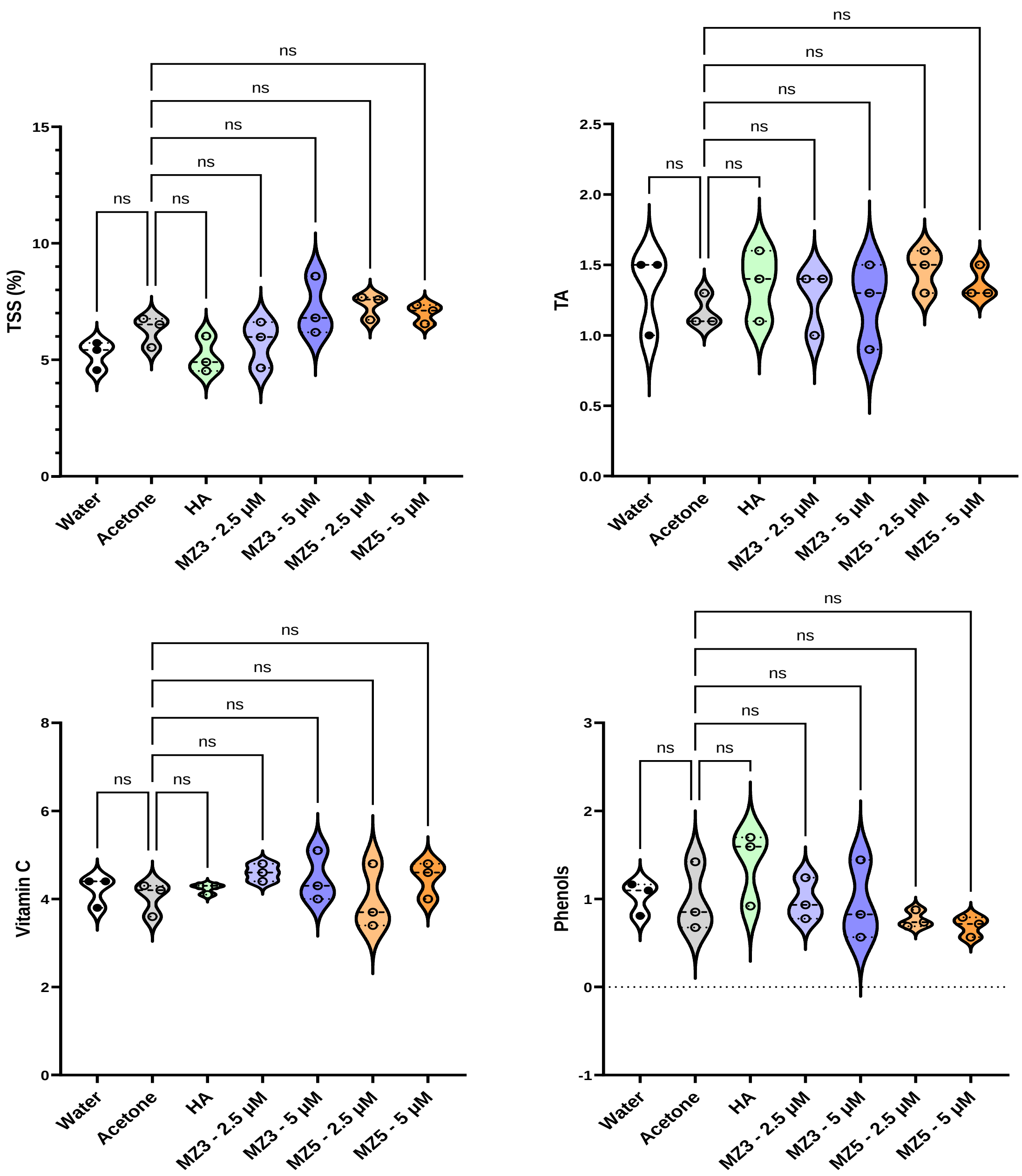

Figure S7. Biochemical analysis of strawberry (cv Sweet Charlie) fruits upon MiZax treatment in the field of KAU, performed in 2022. Data represent mean ± SD. n =3. Statistical analysis was performed using One-way analysis of variance (ANOVA) and Tukey's post hoc test or using two-tail student *t*-test. Asterisks indicate statistically significant differences as compared to mock (**p* < 0.05, ***p*< 0.01, ****p*< 0.001, *****p*< 0.0001; ns, non-significant). HA, humic acid; MZ3, MiZax3; MZ5, MiZax5.

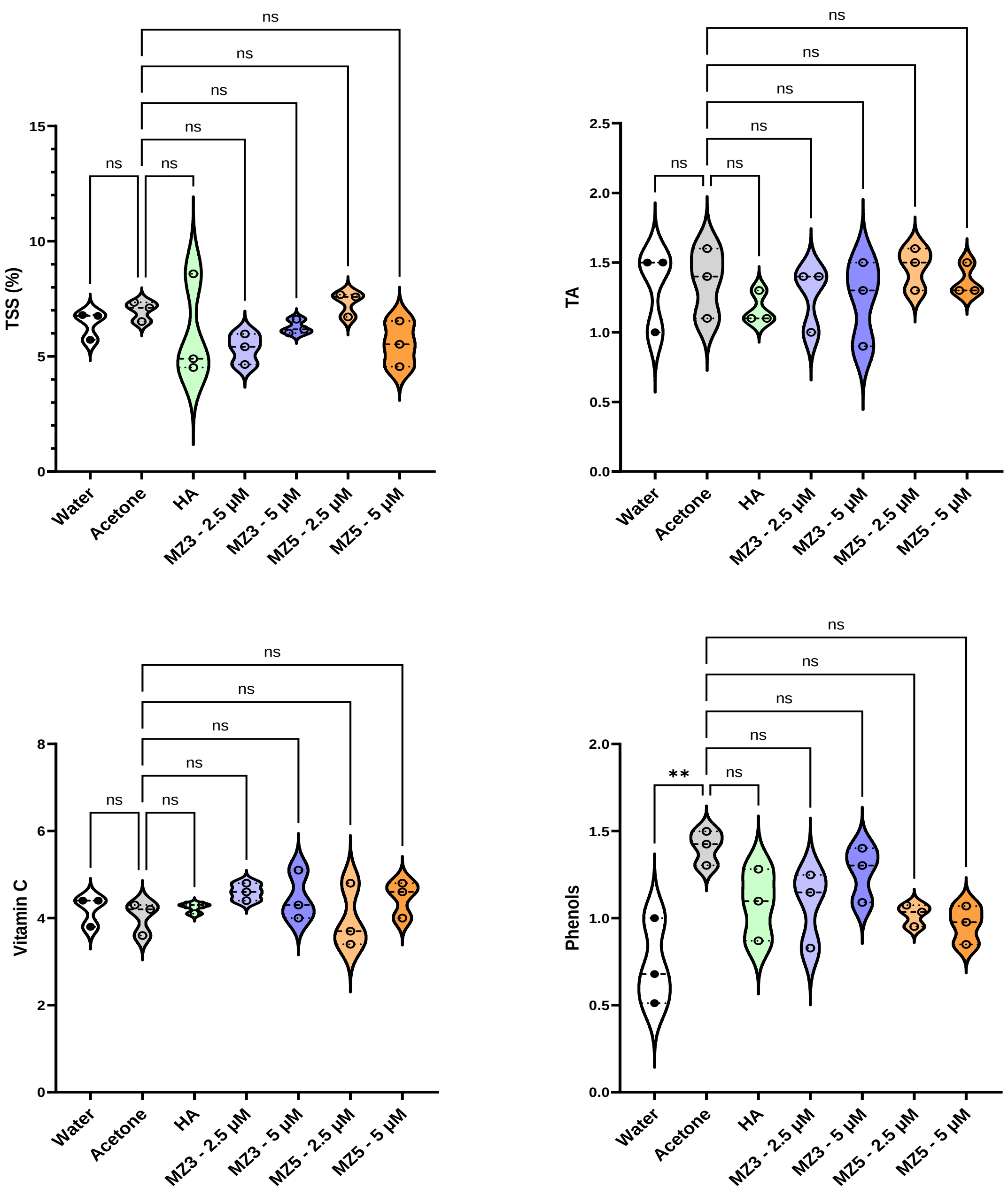

Figure S8. Biochemical analysis of strawberry (cv Festival) fruits upon MiZax treatment in the field of KAU, performed in 2022. Data represent mean ± SD. n =3. Statistical analysis was performed using One-way analysis of variance (ANOVA) and Tukey's post hoc test or using two-tail student *t*-test. Asterisks indicate statistically significant differences as compared to mock (*$p < 0.05$, **$p < 0.01$, ***$p < 0.001$, ****$p < 0.0001$; ns, non-significant). HA, humic acid; MZ3, MiZax3; MZ5, MiZax5.
